# Dual *MGMT* inactivation by promoter hypermethylation and loss of the long arm of chromosome 10 in glioblastoma

**DOI:** 10.1002/cam4.3217

**Published:** 2020-07-14

**Authors:** Sophie Richard, Gaëlle Tachon, Serge Milin, Michel Wager, Lucie Karayan‐Tapon

**Affiliations:** ^1^ Faculté de Médecine Université de Poitiers Poitiers France; ^2^ Laboratoire de cancérologie biologique CHU de Poitiers Poitiers France; ^3^ Laboratoire des Neurosciences Expérimentales et Cliniques INSERM 1084 Poitiers France; ^4^ Laboratoire d’anatomopathologie CHU de Poitiers Poitiers France; ^5^ CHU de Poitiers Poitiers France

**Keywords:** 10q, comparative genomic hybridization, glioblastoma, loss of heterozygosity, MGMT

## Abstract

**Background:**

Epigenetic inactivation of O6‐methylguanine‐methyltransferase (*MGMT*) gene by methylation of its promoter is predictive of Temozolomid (TMZ) response in glioblastoma (GBM). *MGMT* is located on chromosome 10q26 and the loss of chromosome 10q is observed in 70% of GBMs. In this study, we assessed the hypothesis that the dual inactivation of *MGMT*, by hypermethylation of *MGMT* promoter and by loss the long arm of chromosome 10 (10q), may confer greater sensitivity to TMZ.

**Methods:**

A total of 149 tumor samples from patients diagnosed with GBM based on the WHO 2016 classification were included in this retrospective study between November 2016 and December 2018. Methylation status of *MGMT* promoter was evaluated by pyrosequencing and status of chromosome 10q was assessed by array comparative genomic hybridization.

**Results:**

Glioblastoma patients with chromosome 10q loss associated with hypermethylation of *MGMT* promoter had significantly longer overall survival (OS) (*P* = .0024) and progression‐free survival (PFS) (*P* = .031). Indeed, median OS of patients with dual inactivation of *MGMT* was 21.5 months compared to 12 months and 8.1 months for groups with single *MGMT* inactivation by hypermethylation and by 10q loss, respectively. The group with no *MGMT* inactivation had 9.5 months OS. Moreover, all long‐term survivors with persistent response to TMZ treatment (OS ≥ 30 months) displayed dual inactivation of *MGMT*.

**Conclusions:**

Our data suggest that the molecular subgroup characterized by the dual inactivation of *MGMT* receives greater benefit from TMZ treatment. The results of our study may be of immediate clinical interest since chromosome 10q status and methylation of *MGMT* promoter are commonly determined in routine practice.

## Graphical abstract

MGMT can be inactivated by two mechanisms: methylation of promoter and chr10q loss. Dual inactivation of MGMT selected a population with greater benefit from TMZ. Patients with long‐term OS (>30months) presented dual inactivation of MGMT. Chr10q status and methylation of MGMT are commonly determined in routine practice.

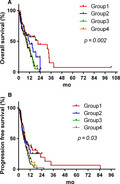

## 1 INTRODUCTION

Glioblastoma (GBM) is the most common and aggressive malignant primary tumor of the central nervous system (CNS) in adults.^1^, ^2^ The therapeutic standard (Stupp's protocol) is currently defined by maximal safe surgical resection followed by radiotherapy plus concomitant alkylating agent temozolomide (TMZ) followed by adjuvant chemotherapy with TMZ.^3^, ^4^, ^5^ However, the response to TMZ varies from one patient to another.^6^

Epigenetic silencing of *MGMT* (*O6‐Methylguanine‐DNA methyltransferase*) by promoter methylation is common in GBM (40%‐50%).^7^ It is predictive of the therapeutic response to alkylating agents such as TMZ, and therefore associated with patient survival.^8^, ^9^ *MGMT* encodes for DNA repair protein, which removes the alkyl groups at the O6‐guanine position induced by alkylating agents. As a result, when not silenced, MGMT neutralizes TMZ cytotoxic action by reducing its therapeutic effect.^10^

The *MGMT* is located at chromosome 10q26.3 and loss of chromosome 10q is frequently observed in GBM (70%).^11^ Despite the importance of 10q loss in gliomagenesis, its association with survival remains controversial. Numerous trials have studied the prognostic impact of 10q loss in GBMs and reported either negative^12^, ^13^, ^14^, ^15^ or neutral ^16^, ^17^, ^18^, ^19^, ^20^ impact on survival.

In tumor cells, the loss of chromosome 10q26.3 implies a loss of heterozygosity (LOH) of *MGMT*. If the promoter of *MGMT* carried by the second allele is hypermethylated, in theory the tumor cells present complete silencing of *MGMT* gene expression. This GBM molecular subtype may present greater sensitivity to TMZ than GBM with *MGMT* inactivation by a single mechanism.

In our study, we aimed to investigate overall survival (OS) and progression‐free survival (PFS) in GBM with dual inactivation of *MGMT* (by methylation of its promoter and chromosome 10q26.3 loss) versus simple inactivation of *MGMT* (by one of the previously cited mechanisms).

## 2 MATERIALS AND METHODS

### 2.1 Study design

We conducted a retrospective study of tumor samples from patients with GBM originating in six different French hospitals. Tumor samples received for routine exploration at the Cancer Biology Department of Poitiers University Hospital between November 2016 and December 2018 were included in this study.

### 2.2 Patients

The study was carried out in accordance with French legislation (French bioethics law No. 2004‐800 of 6 August 2004 and Law No 2012‐300 of 5 March 2012 on research involving the human person) and in accordance with the Helsinki Declaration. Data confidentiality was ensured for all patients.

The study included 149 patients aged ≥ 18 years with confirmed GBM diagnosis by experienced neuropathologists according to the WHO 2016 CNS classification (Table 1). All tumor samples were available for comparative genomic hybridization (CGH) assay and pyrosequencing analysis and 90% of them presented adequate percentage of tumor cells (above the optimum rate of 50%). As our minimum percentage of tumor cells for these techniques was 20%, the remaining samples with rates between ≥ 20% and < 50% were not excluded from the study. One hundred and forty‐two GBM tumors were wild type for *isocitrate dehydrogenase 1/2 genes* (*IDH1/2*), six (4%) were *IDH1* p.R132H‐mutated, and one (0.7%) was *IDH2* p.R172K‐mutated. General features of the cohort such as age, WHO performance status, were collected from the clinical chart.

**TABLE 1:** Demographic, histological, and biological characteristics of patients at inclusion

Features	Group1 (N = 41)	Group2 (N = 27)	Group3 (N = 54)	Group4 (N = 27)	Total (N = 149)	*P*
Demographic data						
Age at diagnosis (y)						.62
Mean	64	62	63	66	64	
Extreme	26‐84	18‐88	32‐82	31‐82	18‐88	
Gender—n (%)						.18
Male	18 (44)	17 (63)	35 (65)	17 (63)	87 (58)	
Female	23 (56)	10 (37)	19 (35)	10 (37)	62 (42)	
WHO performance status—n (%)						.91
0	10 (24)	6 (22)	15 (28)	5 (18)	36 (24)	
1	23 (56)	15 (56)	28 (52)	19 (70)	85 (57)	
2	6 (15)	3 (11)	7 (13)	1 (4)	17 (11)	
3	2 (5)	1 (4)	3 (6)	1 (4)	7 (5)	
4	0	1 (4)	1 (2)	0	2 (1)	
Unknown	0	1 (4)	0	1 (4)	2 (1)	
Histological/Biological data						
Type of specimen—n (%)						.35
Biopsy	16 (39)	16 (59)	22 (41)	13 (48)	67 (45)	
Surgical specimen	25 (61)	11 (41)	32 (59)	14 (52)	82 (55)	
2016 WHO classification—n (%)						.47
GBM *IDH* wild type	38 (93)	25 (92.6)	52 (96)	27 (100)	142 (95)	
GBM *IDH* mutated	3 (7)	2 (7)	2 (4)	0	7 (5)	
*MGMT* methylation						**<.001**
Positive (≥8%)—n (%)	41 (100)	27 (100)	0	0	68 (46)	
Negative (<8%)—n (%)	0	0	54 (100)	27 (100)	81 (54)	
Mean (%)	46	34	3	3	18.5	
10q loss status—n (%)						**<.001**
Positive (including *MGMT*)	41 (100)	0	54 (100)	0	95 (64)	
Negative	0	27 (100)	0	27 (100)	54 (36)	

Note

Significant values are indicated in bold.

### 2.3 Treatment and follow‐up

Every patient in the study received the recommended standard treatment (Stupp's protocol).^3^, ^4^ Tumor progression was determined based on magnetic resonance imaging according to the RANO criteria.^21^ Tumor progression management and second‐line treatment (surgery, radiotherapy and/or chemotherapy) were discussed in multidisciplinary coordination meetings.

### 2.4 Pyrosequencing

All molecular analyses were conducted as routine practice for GBM biomarker testing at the Cancer Department of Poitiers University Hospital (France). Tumor DNA was extracted using the Maxwell® FFPE Tissue LEV DNA kit (AS1130, Promega) from an average of six sections of 10 µm thick fixed paraffin‐embedded tissues.

The methylation profile of five CpG sites, located in the region of + 17 to + 39 of exon 1 of the *MGMT* gene (chromosome 10q26 ranging from 131 265 5007 to 131 265 535) was analyzed. The exact sequence was: 5′‐**CG**GACAG**CG**ATCTCTAA**CGCG**CAAG**CG**CA‐3′. In each series, internal quality control groups were systematically added: a blank and two controls, one highly methylated (MethylatedHuman Control, Promega) and the other unmethylated (UnmethylatedHuman Control DNA, Qiagen). The tumor DNA was bisulfite‐modified using the EZ DNA Methylation‐Gold kit (ZymoResearchn). PCR amplification was performed using 5 µL of bisulfite‐modified DNA using the Pyromark Q24 CpG MGMT^®^ kit (Qiagen) with 1 µL of sense and antisense sequencing primer. Pyrosequencing of *MGMT* PCR products was carried out using PyroMark Q24 Gold Reagents (Qiagen). Finally, the results were interpreted using Pyromark Q24 (Qiagen) software. Representative positive and negative pyrographs are shown in Figure S1. The final methylation percentage was defined as the mean methylation percentage of the five CpG sites. The clinical cutoff for methylation/non‐methylation was set at 8%, an optimal risk cutoff first determined by a retrospective study in 2012^22^ and subsequently confirmed in a prospective study in 2016.^23^

### 2.5 ArrayCGH

This technique was performed on the same extract of DNA used for pyrosequencing exploration; a minimum of 300 ng (37.5 ng/uL) was required. Labeling (Genomic DNA ULS Labeling Kit Agilent), purification, and hybridization of the tumor DNA samples were carried out according to the manufacturer's protocols (Oligonucleotide Array‐Based CGH for Genomic analysis, Agilent). The samples were hybridized with the SurePrint G3 Human CGH Microarray Kit 4 × 180 K. The slides (Hybridization Gasket Slide Kit 4‐pack microarrays Agilent) were analyzed by Agilent SureScan Dx Microarray Scanner Bundle scanner and the TIFF images were obtained using Agilent Scan Control software. Raw data were generated using Feature Extraction software and analyzed by Agilent Cytogenomics software. The main aberration filter was set to call “copy number variation” when at least five consecutive probes deviated from an absolute log^2^ ratio value of 0.25.^24^ All profiles were evaluated by qualified molecular biologists. A representative CGHarray profile with hetorozygous 10q loss is shown in Figure S2.

### 2.6 Statistical analyses

Patients were classified into four groups according to their *MGMT* methylation and 10q26.3 loss status. Comparison of patient characteristics by groups was conducted by chi‐square test for qualitative variables and Kruskal‐Wallis test for quantitative variables. OS and PFS were estimated by the Kaplan‐Meier using the log‐rank test method and were described using median or rate at specific time points along with their 95% confidence interval (CI). For OS, patients known to be alive were censored at the date of their last follow‐up. For PFS, living patients without progression were censored at the date of their last follow‐up. Follow‐up was calculated by a reverse Kaplan‐Meier estimation. Statistical analyses were performed using GraphPadPrism (v6.01) and IBM SPSS Statistics 21 software.

## 3 RESULTS

### 3.1 Patient and tumor characteristics

All in all, 149 GBM specimens were included. Among them, 68 (46%) were *MGMT* hypermethylated and 81 (54%) were *MGMT* unmethylated, 95 (64%) had 10q26.3 loss, and 54 (36%) had no 10q26.3 loss.

**FIGURE 1:**
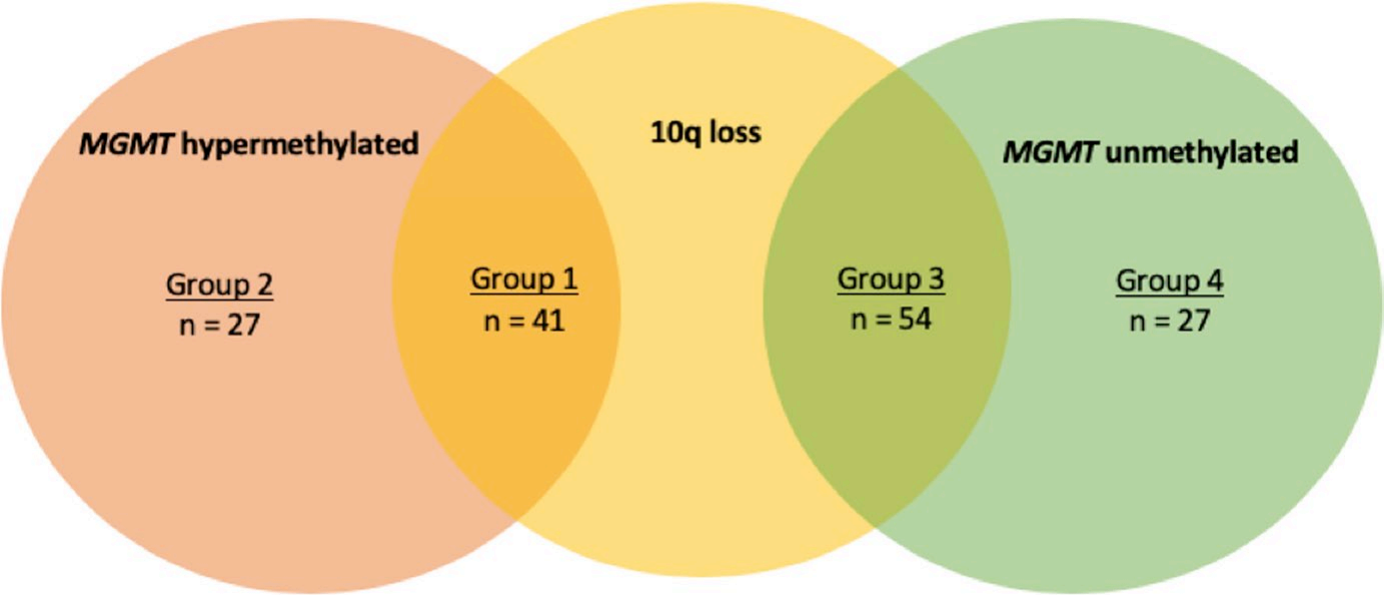
A graphical representation of overlap or lack thereof of the four prognostic groups

Forty‐one tumors (28%) presented dual inactivation of *MGMT* (Group 1: M*GMT* hypermethylated and 10q26.3 loss), 27 tumors (18%) were *MGMT* hypermethylated without 10q26 loss (Group 2), 54 tumors (36%) were *MGMT* unmethylated with 10q26.3loss (Group 3), and 27 tumors (18%) were *MGMT* unmethylated without 10q26.3 loss (Group 4). This distribution is summarized in a graphical representation (Figure 1).

The groups were well balanced with no statistical differences between age, gender, or histobiological data (Table 1).

### 3.2 Treatment delivery

At time of diagnosis, the Stupp's protocol was initiated for all patients starting with surgical intervention. Complete surgery, defined as the absence of visible contrast enhancement on post‐surgery MRI, was possible for only 62 patients (41.6%) (Table 2). One‐hundred and eight patients (72.5%) received 75 mg/m^2^/d TMZ concomitant with radiotherapy delivered at a dose of 60 Gy, distributed in 30 fractions of 1.8‐2 Gy per day, 5 days per week, over a period of 6 weeks. Median time between surgery and radiochemotherapy was 47 days. Among the 108 patients, 87 (80.6%) received adjuvant TMZ at 150‐200 mg/m^2^/d according to the Stupp's protocol. Treatment delivery did not differ between groups except by the number of cycles of adjuvant therapy administrated with more cycles received by patients with hypermethylated and 10q26.3 loss tumor (Group 1, *P* < .001) (Table 2). Sixty‐eight (46%) patients presented tumor progression with no statistical difference between groups (Table S1). Regarding second line treatment, repeat surgery was more frequently performed in patients with dual inactivation of *MGMT* (31%) (*P* = .04).

**TABLE 2:** Therapeutic management of patients at diagnosis

At diagnosis	Group1 (N = 41)	Group2 (N* = 27*)	Group3 (N* = 54*)	Group4 (N* = 27*)	Total (N* = 149*)	*P*
Surgery—n (%)	41 (100)	27 (100)	54 (100)	27 (100)	149 (100)	
Biopsy	16 (39)	16 (59)	22 (41)	13 (48)	67 (45)	.35
Type of surgery	25 (61)	11 (41)	32 (59)	14 (52)	82 (55)	
Complete	20 (80)	7 (64)	26 (81)	9 (64)	62 (76)	.76
Partial	5 (20)	3 (27)	6 (19)	4 (29)	18 (22)	
Unknown	0	1 (9)	0	1 (7)	2 (2)	
Concomitant RT + TMZ n (%)	31 (76)	17 (63)	38 (70)	22 (82)	108 (73)	.45
Adjuvant TMZ—n (%)	27 (66)	15 (56)	30 (56)	15 (56)	87 (58)	.73
TMZ cycles						
≥6 cycles	19 (46)	9 (33)	6 (11)	5 (19)	39 (26)	**<.001**
Median [min‐max]	8.5 [1‐24]	6 [1‐20]	3 [1‐13]	4.5 [1‐14]	5 [1‐24]	**.004**
Supportive care alone—n (%)	6 (15)	6 (22)	10 (19)	3 (11)	25 (17)	.69
TMZ alone—n (%)	4 (10)	3 (11)	6 (11)	1 (4)	14 (10)	.5
RT alone—n (%)	0	1 (4)	0	1 (4)	2 (1)	.31

Note

The median is displayed. Significant values are indicated in bold.

Abbreviations: RT, radiotherapy; TMZ, Temozolomid.

### 3.3 Overall survival and progression‐free survival

After median follow‐up of 18.2 months, 118 patients (79.2%) out of 149 had experienced tumor recurrence and 105 (70.5%) had died. Median OS and median PFS for the whole cohort were 10.2 and 6.4 months, respectively (Figure S3). As expected and as previously described (Hegi et al^8^), patients with *MGMT* hypermethylated tumors had significantly longer OS and PFS than patients with *MGMT* unmethylated tumors (*P* < .001 and *P* = .0054, respectively) (Figure S4A,B). No significant OS/PFS difference was observed between patients with or without 10q26.3 LOH tumors (Figure S4C,D).

**FIGURE 2:**
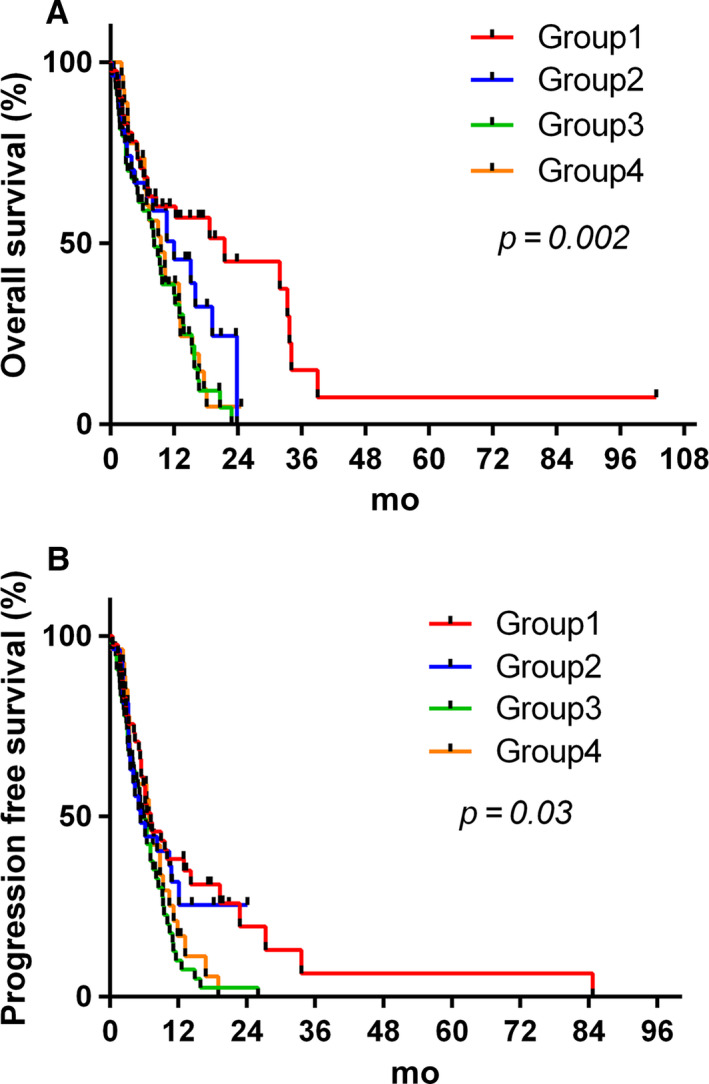
Kaplan‐Meier curves representing OS (A) and PFS (B) according to *MGMT* gene promoter methylation and chromosome 10q status. Group1: *MGMT* hypermethylated and 10q26.3 loss. Group2: *MGMT* hypermethylated without 10q26 loss. Group3: *MGMT* unmethylated with 10q26.3 loss and Group4: *MGMT* unmethylated without 10q26.3 loss

All in all, *MGMT* promoter methylation and 10q26.3 loss status identified four groups of different prognosis. Patients with dual *MGMT* inactivation (Group 1, n = 41) presented the longest OS and PFS with median OS of 21.5 months (*P* = .002) and median PFS of 7.2 months (*P* = .03), with 45% of survivors at 2 years compared to Group 2 (24%), Group 3 (0%), and Group 4 (5%) (*P* < .001) (Table 3, Figure 2A,B). Similarly, Group 1 comprised 31% of patients free of progression after 18 months, compared to Group 2 (25%), Group 3 (3%), and Group 4 (6%). Of particular interest, all long‐term survivor patients (n = 6, 14.6%) with OS ≥ 30 months belonged to Group 1. No patient in the other groups reached this OS. These results remained the same when *IDH* mutated tumors, for which the predictive influence of *MGMT* methylation does not apply, were excluded (n = 142) (Figure S5).

**TABLE 3:** OS and PFS according to the *MGMT* gene promoter methylation and 10q chromosome status in the total study population

Features	Group1 (N = 41)	Group2 (N* = 27*)	Group3 (N* = 54*)	Group4 (N* = 27*)
Median follow up (mo)	16.9	18.2	20.5	24.6
Number of deaths—n (%)	24 (59)	18 (67)	41 (76)	22 (82)
Survival *MGMT* (median—month)	15.1		8.9	
Survival (median—month)	21.5	12	8.1	9.5
Overall survival rate (%)				
6 mo	71	67	61	74
12 mo	60	46	36	39
18 mo	57	33	9	10
24 mo	45	24	0	5
Number of patients with progression—n (%)	32 (78)	16 (59)	46 (85)	24 (88)
Progression‐free survival *MGMT* (median—month)	6.2		6.4	
Progression‐free survival (median—month)	7.2	5.4	6	6.9
Progression‐free survival rate (%)				
6 mo	59	48	49	59
12 mo	38	32	10	17
18 mo	31	25	3	6
24 mo	19	25	3	0

Note

Group1: M*GMT* hypermethylated and 10q26.3 loss. Group2: *MGMT* hypermethylated without 10q26 loss. Group3: *MGMT* unmethylated with 10q26.3 loss and Group4: *MGMT* unmethylated without 10q26.3 loss.

**FIGURE 3:**
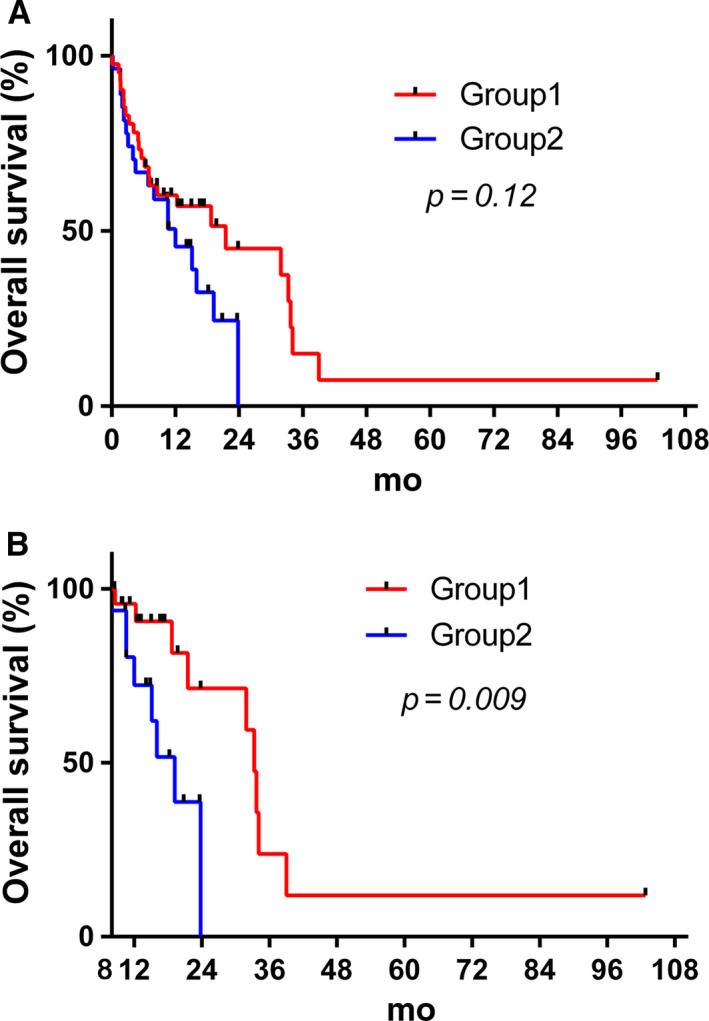
Kaplan‐Meier curves representing OS according to chromosome 10q status in patients with GBM with hypermethylation of the MGMT gene promoter at diagnosis (A) and at 8‐mo follow‐up (B). Group1: MGMT hypermethylated and 10q26.3 loss. Group2: MGMT hypermethylated without 10q26 loss

It is worth noting that OS and PFS were similar during the first 8 months of follow‐up, whatever the molecular profile. In patients with hypermethylation of *MGMT* promoter (n = 68), OS tended to be longer in patients with 10q26.3 loss tumors (Group 1) compared to patients without (Group 2; *P* = .12) (Figure 3A, Figure S6). From 8‐month follow‐up, significantly different OS was observed between these two Groups (*P* = .009; Figure 3B). The hazard ratio of Group 1 with dual inactivation of *MGMT* compared to Group 2 with methylation of *MGMT* alone was 0.33 (95% CI [0.063‐0.604]), which corresponded to a 67% decrease in risk of death. While comparing cases in Group 1 and Group 2, who completed at least six cycles of adjuvant TMZ, OS tended to be statistically different at 8‐month follow‐up (*P* = .06) but not at diagnosis (*P* = .24, Figure S7). However, the number of patient was too low to draw meaningful conclusions (n = 19 and n = 9 respectively).

### 3.4 Univariate and multivariate analysis

Finally, we conducted a uni‐ and multivariate analysis of well‐known markers of interest in GBM in our cohort. Age at diagnosis, the WHO performance status, the extent of surgical resection and dual inactivation of *MGMT* were independent prognostic factors of GBM as they were significantly associated with OS in uni‐and multivariate analysis (Table 4). Age at diagnosis, the WHO performance status, extent of surgical resection and dual inactivation of *MGMT* were also significantly associated with PFS in univariate and remained in multivariate analysis.

**TABLE 4:** Uni‐and multivariate analyses with Cox proportional‐hazards model in the total study population (n = 149) according to OS and PFS

	Univariate analysis			Multivariate analysis		
	*P*	Hazard ratio	CI 95%	*P*	Hazard ratio	CI 95%
Overall Survival						
Gender	.309	0.816	0.552‐1.207			
Age at diagnosis	**<.001**	1.037	1.019‐1.056	**.006**	1.025	1.007‐1.044
WHO	**<.001**	2.078	1.600‐2.699	**<.001**	1.974	1.489‐2.617
Complete surgery	**<.001**	3.028	1.966‐4.665	**<.001**	2.3	1.469‐3.601
Methylation *MGMT* + 10q loss	**.001**	2.306	1.386‐3.834	**.001**	2.411	1.433‐4.054
*IDH*	.319	1.795	0.569‐5.668			
Progression‐Free Survival						
Gender	.593	0.905	0.629‐1.303			
Age at diagnosis	**.001**	1.026	1.011‐1.042	**.045**	1.016	1.000‐1.032
WHO	**<.001**	1.813	1.414‐2.325	**<.001**	1.772	1.348‐2.329
Complete surgery	**<.001**	2.139	1.445‐3.166	**.01**	1.695	1.132‐2.537
Methylation *MGMT* + 10q loss	**.023**	1.639	1.070‐2.512	**.022**	1.67	1.078‐2.588
*IDH*	.361	1.52	0.619‐3.736			

Abbreviation: CI, confidence interval, Significant values are indicated in bold.

## 4 DISCUSSION

In our study, we investigated OS and PFS in GBM according to *MGMT* promoter methylation profile and chromosome 10q status and showed that the combination chromosome 10q26 loss with hypermethylation of the *MGMT* promoter in patients with GBM is an interesting prognostic tool associated with longer OS (*P* = .002) and PFS (*P* = .03). Knowledge of this dual inactivation of *MGMT* can enable selection of long‐term survivor patients (OS ≥ 30 months).

Our population was representative of classic GBM population and no major selection bias was noted. The mean age at GBM diagnosis was 64 years with an M/F ratio of 1.4 in agreement with epidemiological studies.^1^, ^2^, ^25^ Our cohort consisted of 95% primary GBMs and 5% secondary GBMs, which was consistent with the literature.^26^ Among the latter *IDH1* R132H was the most frequent mutation (86%). Hypermethylation of *MGMT* promoter was present in 46% of GBMs and chromosome 10q26.3 loss in 64%. Taken together, these observations corroborated the literature.^27^, ^28^

All patients initiated the Stupp protocol by undergoing surgical procedures. Among them, 73% received concomitant radiochemotherapy after surgical procedure and 58% received adjuvant TMZ. Twenty‐six percent received the complete standard treatment (surgery, concomitant radiochemotherapy followed by at least six cycles of adjuvant TMZ). Patients who were not able to receive radiochemotherapy (27%) could instead receive either TMZ alone (9%), or radiotherapy alone (1%) or supportive cares alone (17%). These results were similar to the 2005 Stupp et al study in which only 85% of patients had received radiochemotherapy post‐surgery and only 36.6% had received the complete standard treatment.^3^ As in the literature, we reported age at diagnosis, WHO performance status and extent of surgical resection as independent prognostic factors.^8^, ^29^, ^30^ We did not find any prognostic impact of chromosome 10q loss by itself in our cohort. Data in the literature are conflicting with chromosome 10q loss, sometimes described as a poor prognostic factor^12^, ^13^, ^14^, ^15^ and sometimes as a non‐impact.^16^, ^17^, ^18^, ^19^, ^20^

Median OS of the cohort was short, 10.2 months, compared to the Stupp et al standard 14.6 months. One explanation could come from the higher number of patients with only biopsy instead of complete resection, 45% compared to 16% in the Stupp et al study. As biopsy resection is known to be a negative prognosis marker compared to complete surgery,^31^, ^32^ it is possible that the high percentage of biopsy explains the relatively low median OS of our study. We have no explanation for this high rate of biopsy but it did not cause major bias as the number of biopsies was equally distributed between groups (*P* = .76). Another explanation for the low OS could come from the median delay between surgery and radiotherapy, which was slightly longer than recommended, 47 instead of 42 days (Referential “Association des neuro‐oncologues d'expression francaise” 2018). However, the influence of this delay on survival is controversial. An overly lengthy delay would be deleterious, or without influence and even beneficial, depending on studies.^33^, ^34^, ^35^, ^36^, ^37^

When focusing on molecular aspects, patients with GBM with dual mechanisms of *MGMT* inactivation had longer OS (*P* = .002) and PFS (*P* = .03). In the hypermethylated group (Group 1 + 2; n = 68), patients with loss of chromosome 10q had longer OS from 8‐month follow‐up than patients without 10q loss (*P* = .009). These results were consistent with the Hegi et al study,^8^ in which it was also observed that OS did not differ according to *MGMT* promoter methylation status during the first 9 months of follow‐up. As a result, even though the MGMT promoter methylation is significantly correlated with TMZ response, during the first months of therapeutic management it does not provide reliable prognostic information, whatever the chr10q status of the GBM patients. In GBM studies such as ours, patients can be included at an advanced stage of disease or have altered general state of health, which means that their immediate survival may no longer depend on underlying molecular mechanisms, for example, *MGMT* methylation status, but rather on other prognostic factors such as age, WHO performance status, co‐morbidities or surgical management. Bady et al also investigated the interaction between 10q deletion and *MGMT* methylation and found no significant association (*P* = .196) in a TCGA‐Glioma‐II/III data set.^38^ Another team investigated the association between *MGMT* mRNA expression and chr10 copy number and showed a lack of significant differences between cases with chromosome 10 monosomy, *MGMT* locus deletion or normal copy number.^39^ However, they did not correlate their results with survival data, and the techniques used for *MGMT* methylation and copy number determination were less sensitive. Finally, another study found no influence of 10q LOH over OS independently of *MGMT* methylation status, but it was performed on a cohort of mix GBM and low grade gliomas.^40^

We observed no PFS differences (*P* = .79) between group 1 and group 2, probably due to the fact that the majority of patients had tumor progression within the first 8 months of follow‐up. This lack of association can also be explained by the inherent difficulties of determination of true tumor progression, distinguished from pseudoprogression and radionecrosis.^41^ Besides, pseudoprogression is more likely to occur in patients with methylation of the *MGMT* promoter.^42^, ^43^

It is of interest to note that patients with dual *MGMT* inactivation received a higher number of adjuvant TMZ cycles (*P* < .001) during therapeutic management at diagnosis and during revision surgery at tumor progression (*P* = .04). A more intensive treatment might also explain why they lived longer. However, according to our hypothesis, this difference of therapeutic management could in fact be the reflection of the better prognosis of patients from group 1. Indeed, dual inactivation of *MGMT* may increase sensitivity of GBM patients to TMZ treatment and could, therefore, result in a greater number of adjuvant TMZ cures. Due to their maintained general health condition, these patients would then benefit from more frequent revision surgery on tumor progression. Of the 16 patients in this group who relapsed, five (31%) underwent a new surgical procedure for tumor progression compared to only four (15%) out of the 27 patients in the unmethylated group with 10q loss (Group 3) and none in the other two groups. After detailed study of these five patients, it appeared that they all had OS ≥ 30 months. In addition, we observed that among the 41 patients with dual inactivation of *MGMT*, six (15%) survived ≥ 30 months, whereas no patient in the other groups in the study reached this survival time.

One of our study limitations was the lack of statistical power in the hypermethylated group, which allowed us to highlight the interest of 10q loss not from diagnosis but only from 8‐month follow‐up. This lack of power could be explained by the small size of our cohort (149 with only 27 patients in each group with no 10q loss), linked to technical limitations. Many samples were not eligible for the study due to inconclusive onco‐biological results. Among the 259 patients eligible for the study, 110 (42.5%) were excluded: 93 had non‐contributory CGHarray analyses, nine had non‐contributory pyrosequencing analyses and eight had non‐contributory analyses for both techniques. Indeed, quality and, most of all, quantity of tumor DNA extracted from fixed embedded paraffin tissue was frequently insufficient for CGH exploration. However, the use of CGH to study 10q loss is a key feature of our study as most other trials on this subject used LOH analysis by microsatellite markers.^13^, ^14^, ^16^, ^17^, ^19^ Despite the need for a large amount of tumor DNA, array CGH in routine practice offers significant advantages over LOH as it is a genome‐wide screening technique that can detect deletion of chromosome 10q along with gain of chromosome 7 in GBM and it can also be useful to diagnose oligodendroglioma by detecting 1p19q co‐deletion at high resolution. Another limitation of our study is the lack of validation cohort, which would have ascertained our results. Other studies on larger cohorts have previously been conducted, such as The Cancer Genome Atlas (TCGA) or the Chinese Glioma Genome Atlas (CGGA), and the methylation of *MGMT* promoter and the copy number status were part of the data they gathered. However, they did not address the specific question of correlation between 10q loss and *MGMT* methylation, as we did. While looking at TCGA dataset, the number of patients without 10q loss was too low to perform the same analysis, and to draw reliable conclusions. Maybe, they did not use the same technique for copy number determination as ours.

To conclude, the 10q loss associated with hypermethylation of *MGMT* could be identified as a theranostic molecular signature of GBM, enabling selection of patients for whom TMZ was most likely to be beneficial. Given the increasingly systematic nature of the study of chromosome 10q status in integrated histopathological and molecular diagnosis of the WHO 2016 classification, combined with highly recommended study of *MGMT* methylation status, this signature could easily be incorporated into GBM biological and clinical routine. Finally, further prospective study that would include adequately treated patients only (patients who have completed Stupp protocol with at least six cycles of adjuvant TMZ) would provide even more insight on the true prognostic benefit of dual inactivation of *MGMT*.

## CONFLICT OF INTEREST

The authors have no conflict of interest related to this work.

## AUTHOR CONTRIBUTIONS

All cited authors have made substantial contributions to conception and design, or acquisition of data, or analysis and interpretation of data. They have been involved in drafting the manuscript or revising it critically for important intellectual content; They all have given final approval of the version to be published. They have agreed to be accountable for all aspects of the work in ensuring that questions related to the accuracy or integrity of any part of the work are appropriately investigated and resolved.

## Supporting information

Fig S1

Fig S2

Fig S3

Fig S4

Fig S5

Fig S6

Fig S7

Table S1

## Data Availability

The data that support the findings of this study are available from the corresponding author upon reasonable request.
