# Supplementary material for: Dual MGMT inactivation by promoter hypermethylation and loss of the long arm of chromosome 10 in glioblastoma
Source: Cancer Med. 2020 Jul 14;9(17):6344–53. doi: 10.1002/cam4.3217 (PMC7476845; doi:10.1002/cam4.3217)
Supplement: Supplementary file 1 — Fig S1 [file CAM4-9-6344-s001.pdf]

**Well: B2**
Assay: MGMT
Sample ID: MGMT 961
Note:
Analysis version: 2.0.7

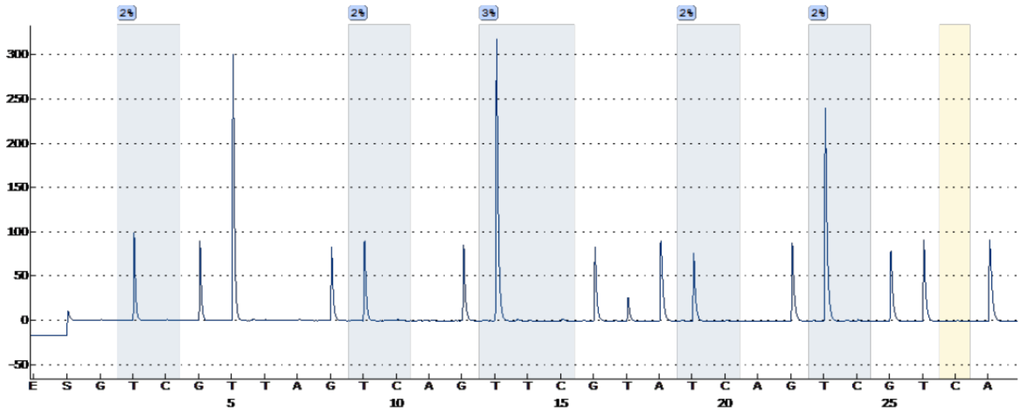

Sequence to analyze:
YGTTTTGYGTTTYGAYGTTYGTAGGTTTT

| Position | 1 | 2 | 3 | 4 | 5 |
|---|---|---|---|---|---|
| Quality | Passed | Passed | Passed | Passed | Passed |
| Meth (%) | 2 | 2 | 3 | 2 | 2 |

No warnings.

**Well: A7**
Assay: MGMT
Sample ID: MG 1106
Note:
Analysis version: 2.0.7

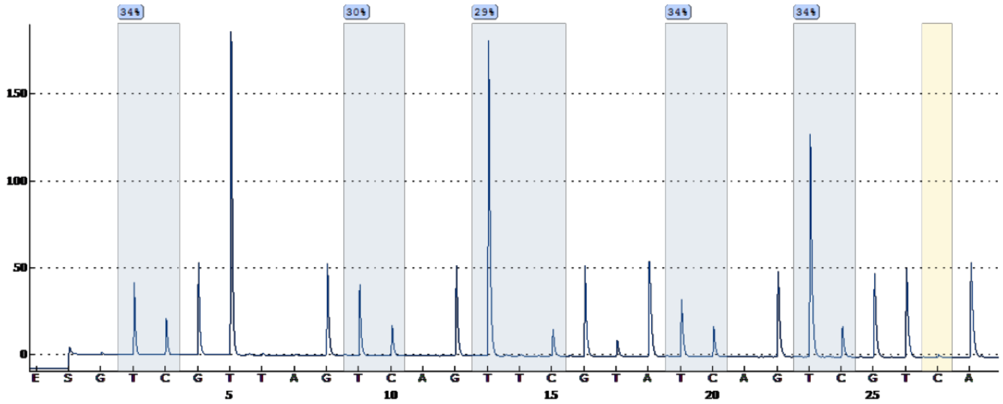

Sequence to analyze:
YGTTTTGYGTTTYGAYGTTYGTAGGTTTT

| Position | 1 | 2 | 3 | 4 | 5 |
|---|---|---|---|---|---|
| Quality | Passed | Passed | Passed | Passed | Passed |
| Meth (%) | 34 | 30 | 29 | 34 | 34 |

No warnings.

**<u>Suppl. Figure 1:</u>** Pyrographs showing the methylation percentage of the 5 CpG sites of MGMT promoter of A) a methylated sample B) an unmethylated sample.
