# Supplementary figures and images for: Dual MGMT inactivation by promoter hypermethylation and loss of the long arm of chromosome 10 in glioblastoma
Source: Cancer Med. 2020 Jul 14;9(17):6344–53. doi: 10.1002/cam4.3217 (PMC7476845; doi:10.1002/cam4.3217)

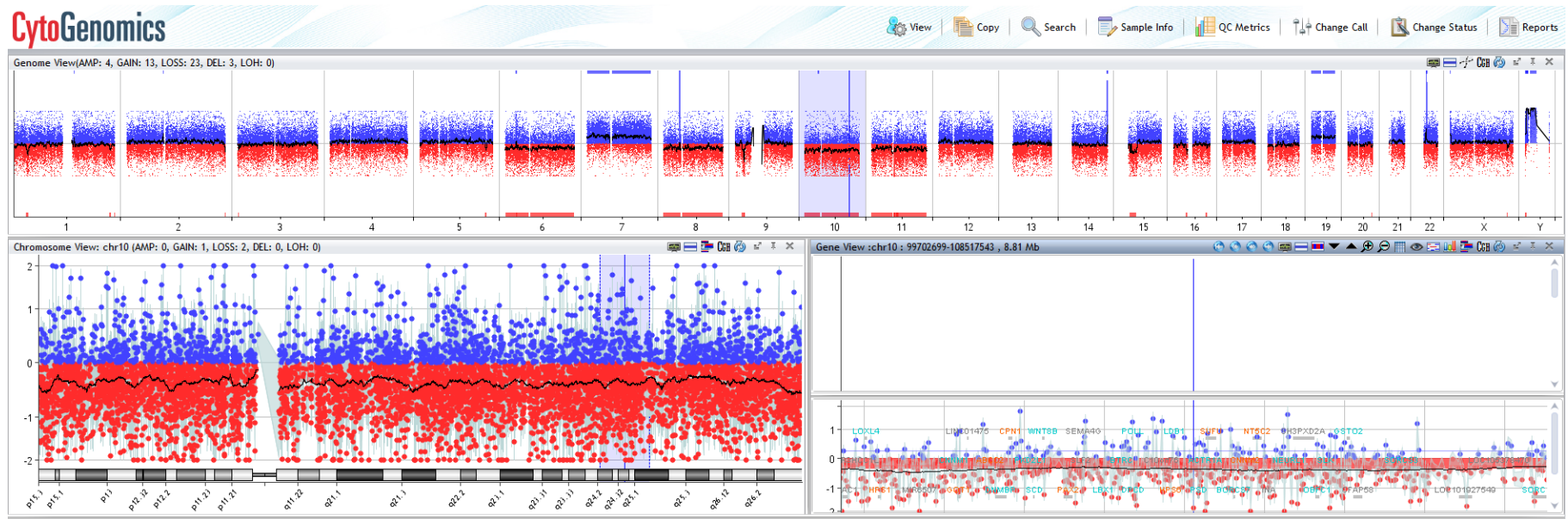

**Suppl. Figure 2:** CGHarray profile of a tumor with heterozygous loss of 10q (log ratio = -0.36).

Supplement: Supplementary file 2 — Fig S2 [file CAM4-9-6344-s002.pdf]

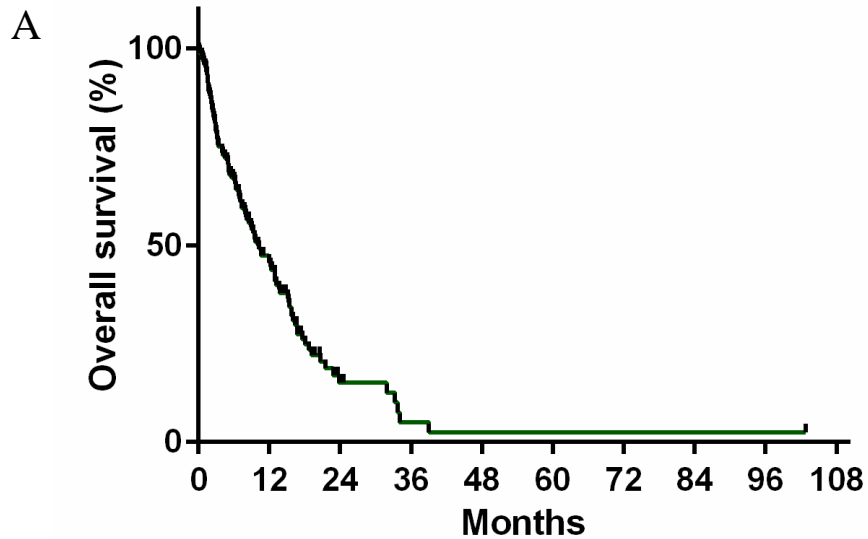

B

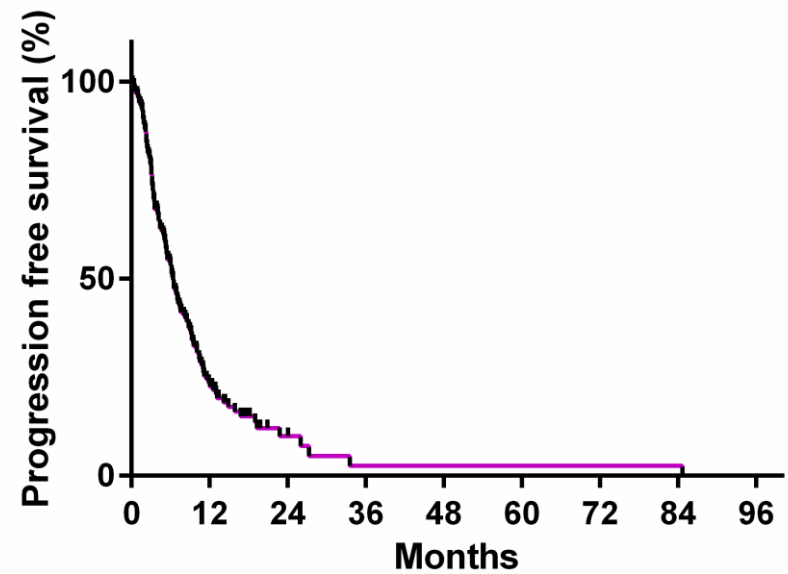

**Suppl. Figure 3:** Kaplan Meier curves representing OS (A) and PFS (B) for the whole cohort.

Supplement: Supplementary file 3 — Fig S3 [file CAM4-9-6344-s003.pdf]
