# Supplementary material for: Dual MGMT inactivation by promoter hypermethylation and loss of the long arm of chromosome 10 in glioblastoma
Source: Cancer Med. 2020 Jul 14;9(17):6344–53. doi: 10.1002/cam4.3217 (PMC7476845; doi:10.1002/cam4.3217)
Supplement: Supplementary file 4 — Fig S4 [file CAM4-9-6344-s004.pdf]

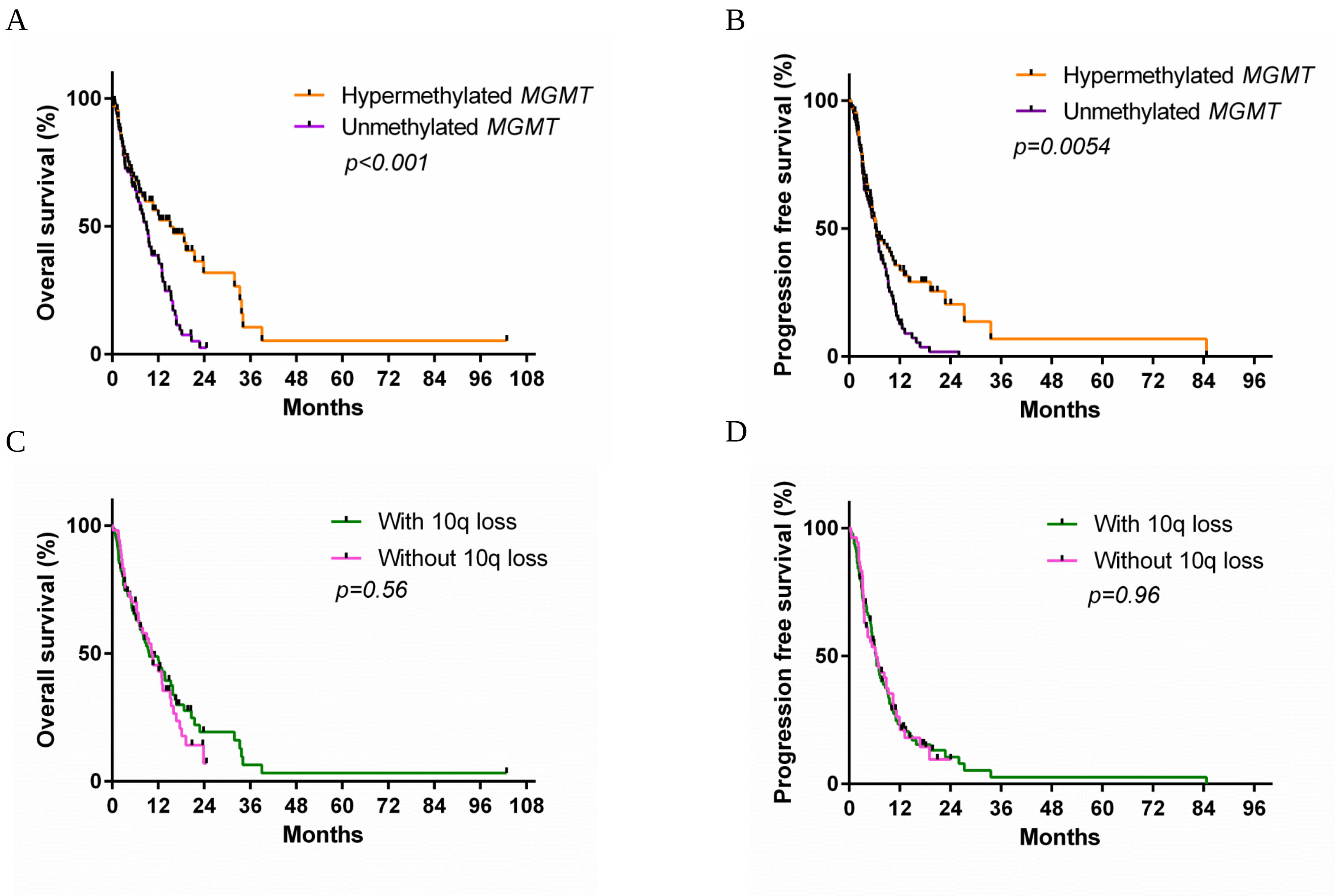

Suppl. Figure 4: Kaplan Meier curves representing OS (A) and PFS (B) according to *MGMT* gene promoter methylation status and according to 10q loss (C-D).
