# Supplementary material for: Dual MGMT inactivation by promoter hypermethylation and loss of the long arm of chromosome 10 in glioblastoma
Source: Cancer Med. 2020 Jul 14;9(17):6344–53. doi: 10.1002/cam4.3217 (PMC7476845; doi:10.1002/cam4.3217)
Supplement: Supplementary file 5 — Fig S5 [file CAM4-9-6344-s005.pdf]

A

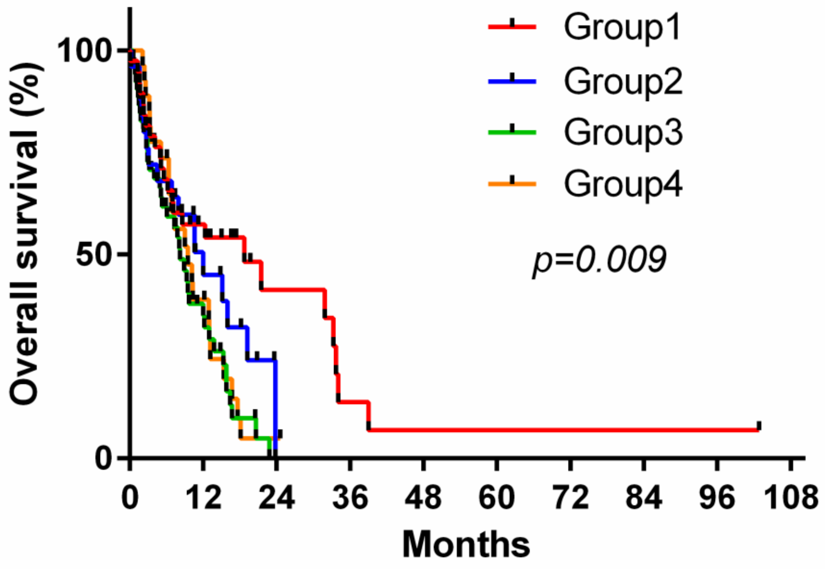

B

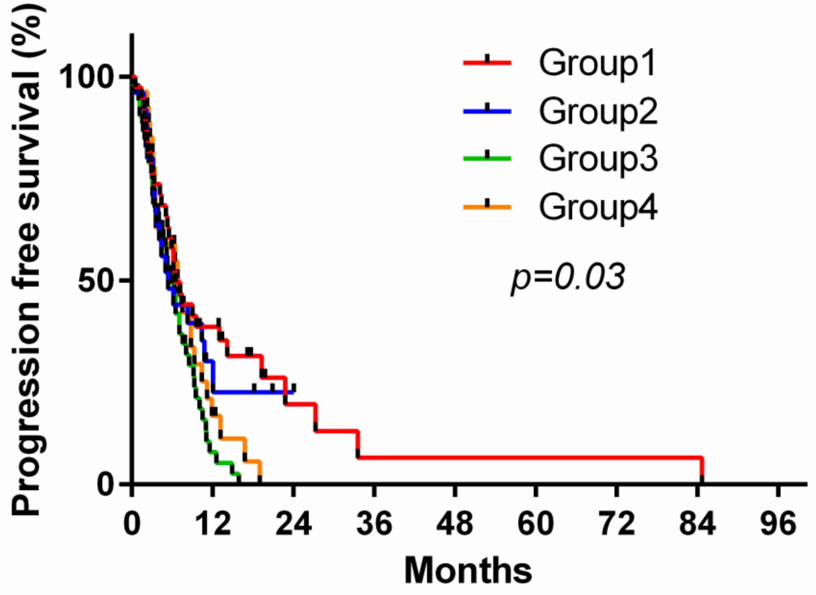

Suppl. Figure 5: Kaplan Meier curves representing OS (A) and PFS (B) according to *MGMT* gene promoter methylation and chromosome 10q status in patients without *IDH* mutation.

Group1: MGMT hypermethylated and 10q26.3 loss. Group2: MGMT hypermethylated without 10q26 loss. Group3: MGMT unmethylated with 10q26.3 loss and Group4: MGMT unmethylated without 10q26.3 loss.
