# Supplementary material for: Dual MGMT inactivation by promoter hypermethylation and loss of the long arm of chromosome 10 in glioblastoma
Source: Cancer Med. 2020 Jul 14;9(17):6344–53. doi: 10.1002/cam4.3217 (PMC7476845; doi:10.1002/cam4.3217)
Supplement: Supplementary file 6 — Fig S6 [file CAM4-9-6344-s006.pdf]

A

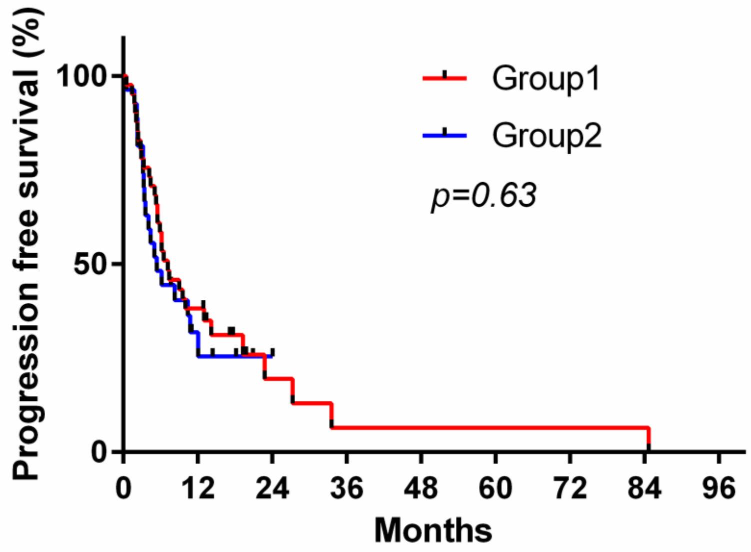

B

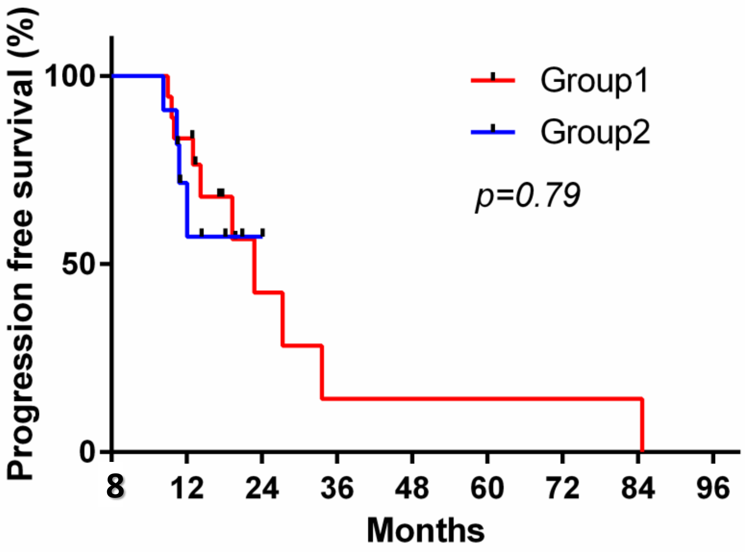

<u>Suppl. Figure 6:</u> Kaplan Meier curves representing PFS according to chromosome 10q status in patients with GBM with hypermethylation of *MGMT* promoter gene from treatment initiation (A) and at 8 month follow-up (B).
Group1: *MGMT* hypermethylated and 10q26.3 loss. Group2: *MGMT* hypermethylated without 10q26 loss
