# Supplementary material for: Dual MGMT inactivation by promoter hypermethylation and loss of the long arm of chromosome 10 in glioblastoma
Source: Cancer Med. 2020 Jul 14;9(17):6344–53. doi: 10.1002/cam4.3217 (PMC7476845; doi:10.1002/cam4.3217)
Supplement: Supplementary file 7 — Fig S7 [file CAM4-9-6344-s007.pdf]

A

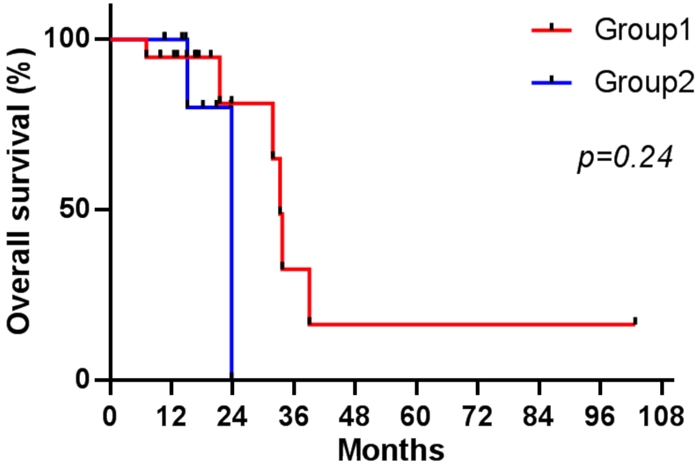

B

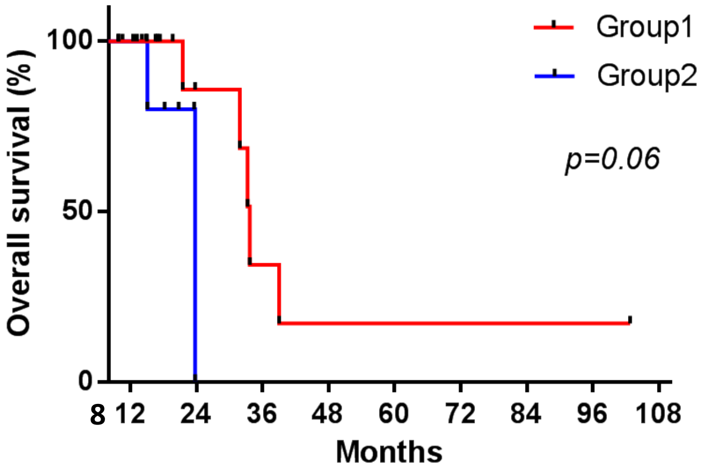

Suppl. Figure 7: Kaplan Meier curves representing OS for Group1 and Group2 who completed at least 6 cycles of adjuvant TMZ at diagnosis (A) and at 8-month follow-up (B).
