# Supplementary material for: Dual MGMT inactivation by promoter hypermethylation and loss of the long arm of chromosome 10 in glioblastoma
Source: Cancer Med. 2020 Jul 14;9(17):6344–53. doi: 10.1002/cam4.3217 (PMC7476845; doi:10.1002/cam4.3217)
Supplement: Supplementary file 8 — Table S1 [file CAM4-9-6344-s008.pdf]

<u>Supplementary Table 1:</u> Therapeutic management of patients at 1st tumor progression. Some patients had more than one treatment.

**At tumor progression *N*(%):**	**Group1 *N=16* (39)**	**Group2 *N=10* (37)**	**Group3 *N=27* (50)**	**Group4 *N=15* (56)**	**Total *N=68* (46)**	*p=0.37*
Second surgery - *n*(%)	5 (31)	0	4 (15)	0	9 (13)	***0.04***
Second line of RT - *n*(%)	2 (13)	1 (10)	0	0	3 (4)	*0.16*
Second line chemotherapy - *n*(%)	14 (88)	4 (40)	18 (67)	10 (67)	46 (68)	*0.09*
Bevacizumab+Lomustine	8 (57)	4 (100)	14 (78)	8 (80)	34 (74)	
Bevacizumab	1 (7)	0	2 (11)	0	3 (7)	
Lomustine	1 (7)	0	1 (6)	1 (10)	3 (7)	
Bevacizumab+TMZ	1 (7)	0	1 (6)	0	2 (4)	
TMZ intensification	2 (14)	0	0	1 (10)	3 (7)	
Bevacizumab+Irinotecan	1 (7)	0	0	0	1 (2)	
Supportive care alone - *n*(%)	2 (13)	6 (60)	8 (30)	5 (33)	21 (31)	*0.09*

RT: radiotherapy, TMZ: Temozolomid
